# Supplementary figures and images for: The cell surface hyaluronidase TMEM2 plays an essential role in mouse neural crest cell development and survival
Source: PLoS Genet. 2022 Jul 15;18(7):e1009765. doi: 10.1371/journal.pgen.1009765 (PMC9328550; doi:10.1371/journal.pgen.1009765)

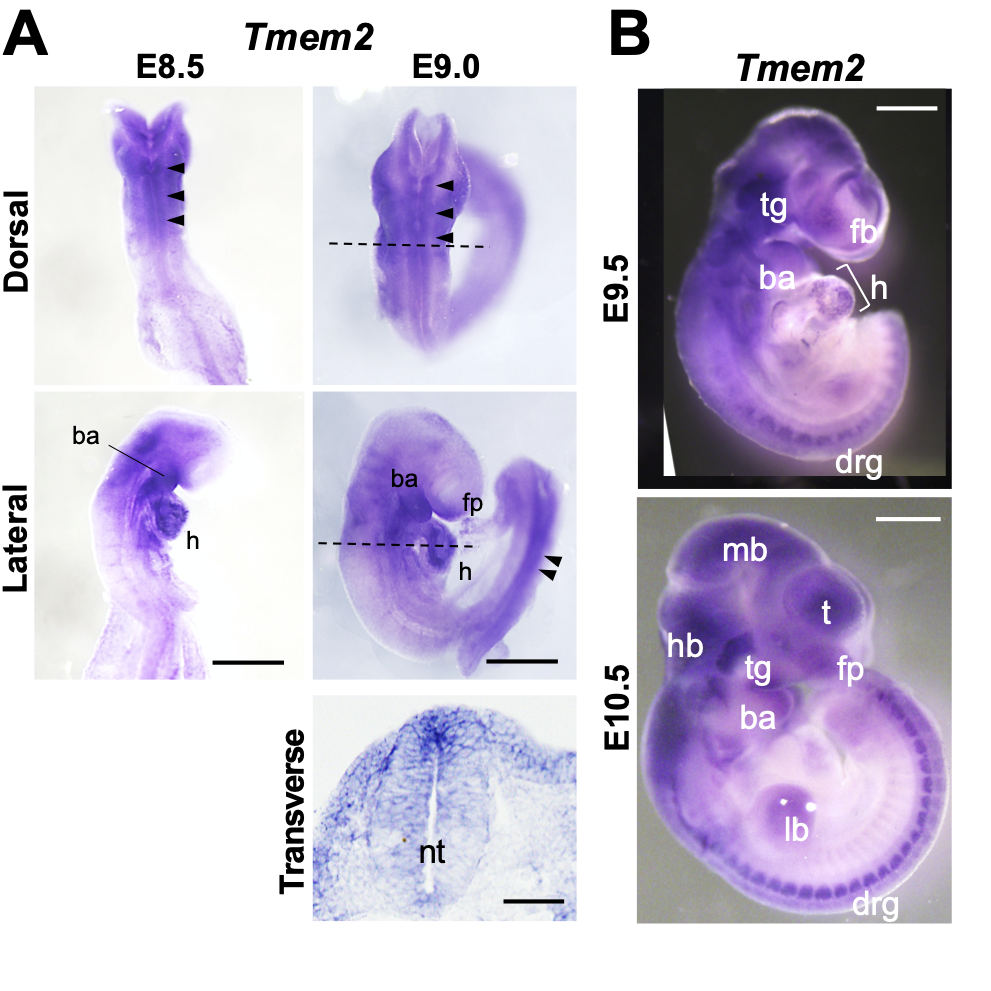

Supplement: S1 Fig — (A) Whole-mount images of the dorsal and lateral aspects of embryos at E8.5 and E9.0 and a transverse section through the neural tube at E9.0. Robust Tmem2 expression is observed in the dorsal midline region of the neural tube (arrowheads), the facial prominence, the branchial arches, and the heart. (B) Whole-mount in situ hybridization images at E9.5 and E10.5. ba, branchial arch; drg, dorsal root ganglia; fb, forebrain; fp, facial prominence; h, heart; hb, hindbrain; mb, midbrain; tg, trigeminal ganglia. Scale bars, 250 μm in A; 500 μm in B. (TIFF) [file pgen.1009765.s001.tiff]

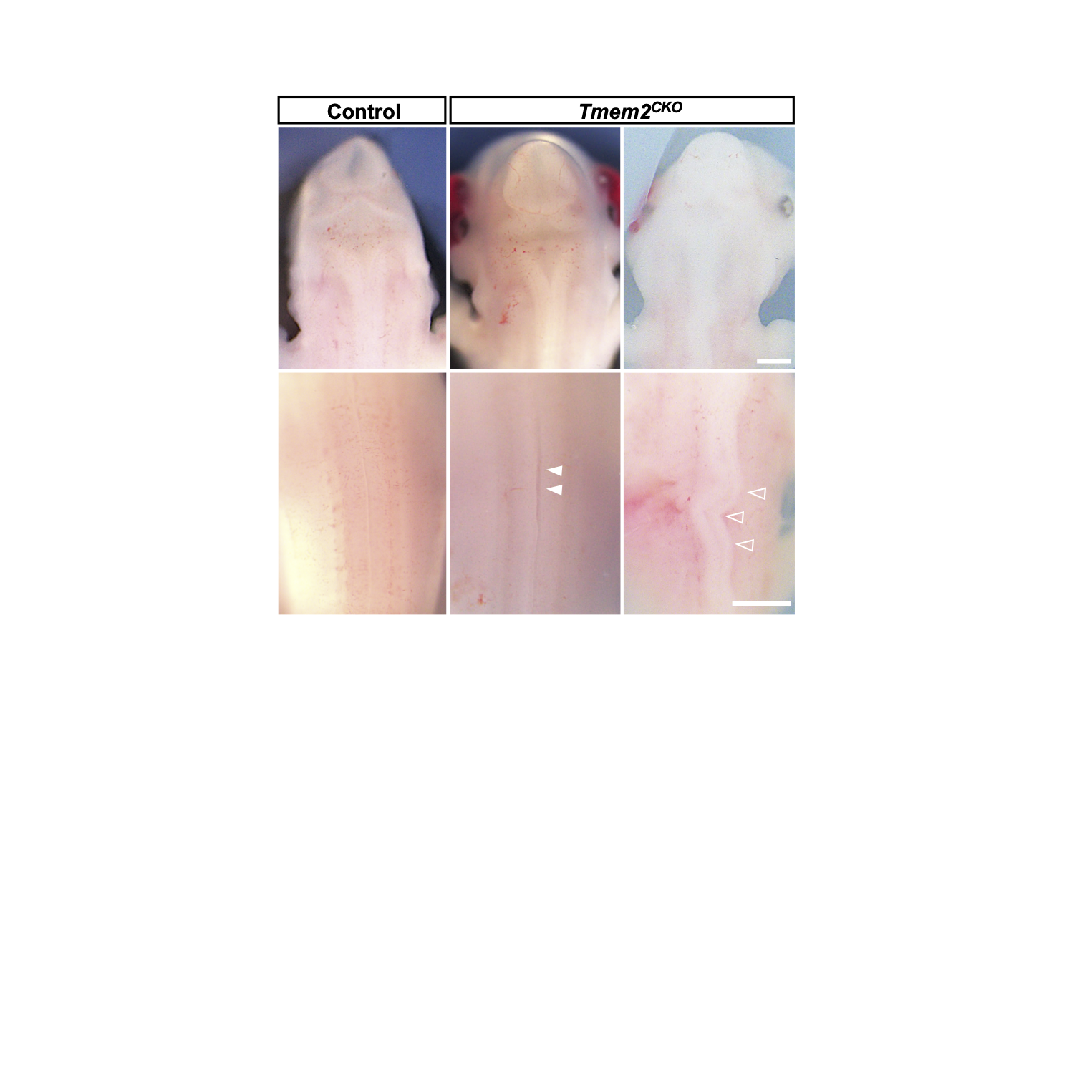

Supplement: S2 Fig — Images show dorsal views of Tmem2CKO and control embryos at E12.5. A fraction (4 of 42, 9.5%) of Tmem2CKO embryos exhibit defects in the neural tube, including incomplete neural tube closure (filled arrowheads) and kinking of the neural tube (open arrowheads). Scale bar, 500 μm. (TIFF) [file pgen.1009765.s002.tiff]

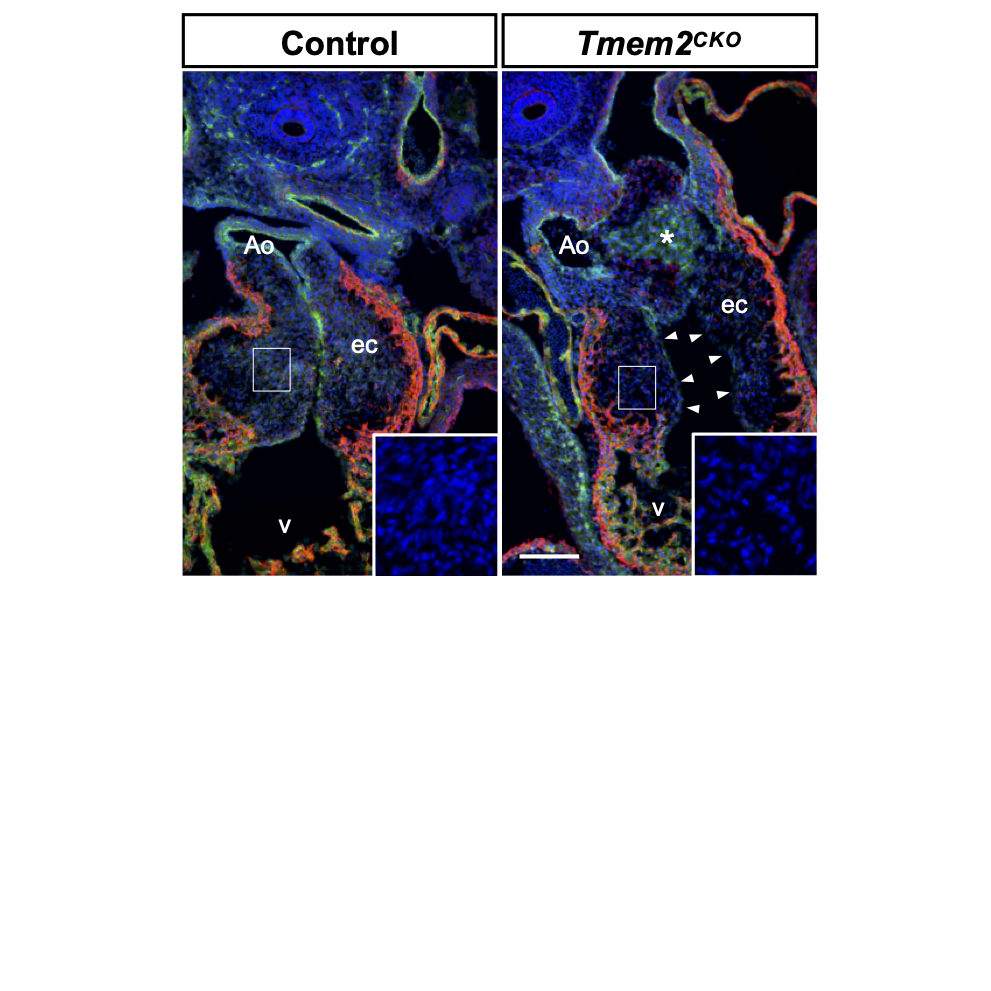

Supplement: S3 Fig — Transverse sections through the outflow tract region of E12.5 Tmem2CKO and control embryos were stained with anti-CD31 (green), anti-αSMA (red), and DAPI (blue). Asterisk indicates abnormal aggregates of endocardial cells in endocardial cushion mesenchyme. Arrowheads in the Tmem2CKO embryo point to lack of normal endocardial layer overlying the conotruncal endocardial cushions. Insets show enlarged images of the endocardial cushion mesenchyme. Ao, aorta; ec, endocardial cushion; v, ventricle. Scale bar, 200 μm. (TIFF) [file pgen.1009765.s003.tiff]

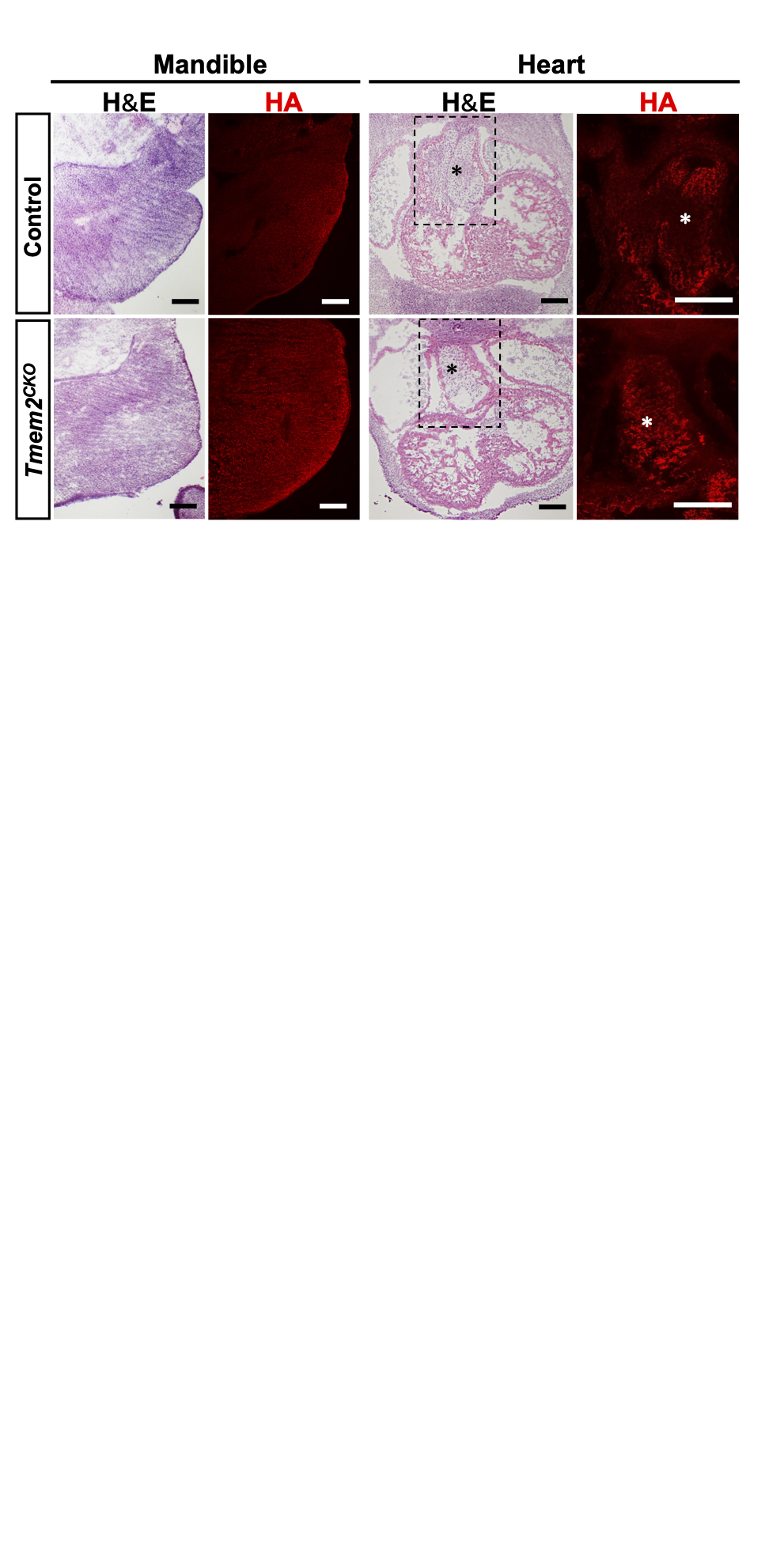

Supplement: S4 Fig — Transverse sections of the mandible and the heart were stained with H&E or labeled with bHABP (red). Aberrant HA accumulation is observed in the mandible and the heart (areas indicated by rectangles are enlarged). Asterisks indicate endocardial cushion mesenchyme. Scale bar, 250 μm. (TIFF) [file pgen.1009765.s004.tiff]

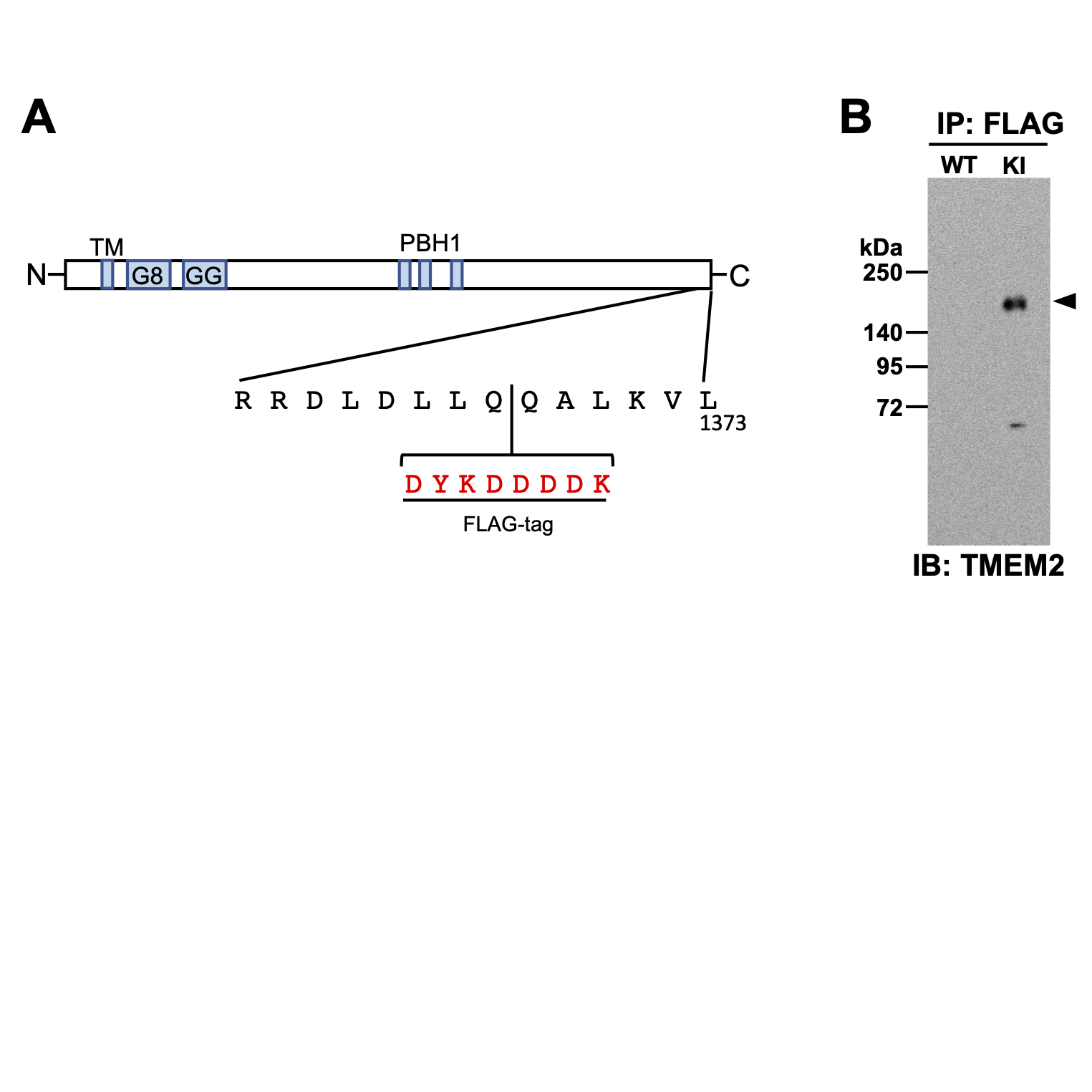

Supplement: S5 Fig — (A) Schematic diagram of FLAG-tagged TMEM2 protein expressed from the Tmem2-FLAGKI locus. (B) Expression of TMEM2-FLAG protein in Tmem2-FLAGKI mice. TMEM2-FLAG protein was immunoprecipitated from the lysate of E11.0 whole embryos with anti-FLAG M2 antibody (IP). Precipitated materials were subjected to immunoblotting analysis (IB) with anti-TMEM2 antibody. (TIFF) [file pgen.1009765.s005.tiff]

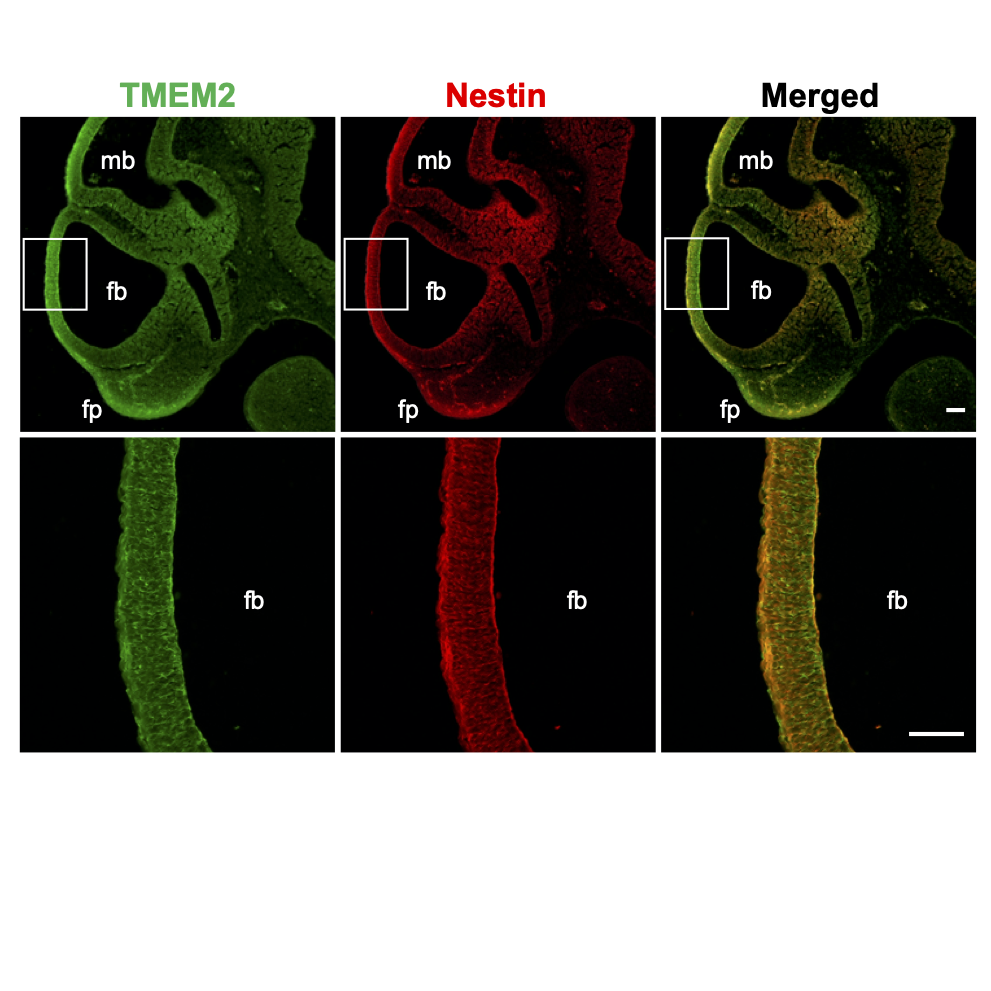

Supplement: S6 Fig — Sagittal sections of Tmem2-FLAGKI reporter embryos at E11.0 were double-labeled with anti-FLAG (to detect TMEM2-FLAG protein) and anti-Nestin (to label neuroepithelial cells) antibodies. Areas indicated by boxes are enlarged in lower panels. fb, forebrain; fp, facial prominence; mb, midbrain. Scale bar, 50 μm. (TIFF) [file pgen.1009765.s006.tiff]

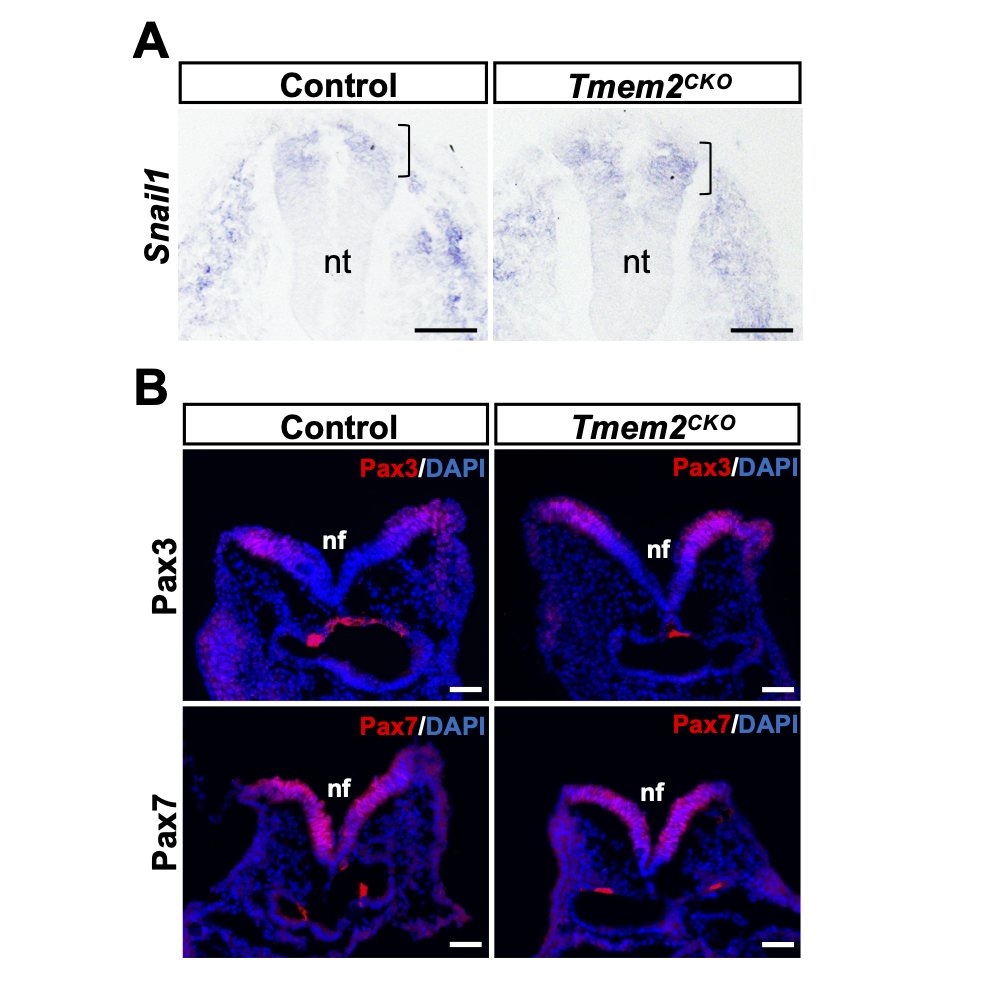

Supplement: S7 Fig — (A) In situ hybridization in transverse sections shows that Snail1 expression is detected in the dorsal neural tube (brackets) of control and Tmem2CKO embryos at E8.5. Scale bar, 250 μm. (B) Transverse sections of control and Tmem2CKO embryos at E8.0 were labeled with anti-Pax3 or anti-Pax7 antibody. Scale bars, 100 μm. nt, neural tube; nf, neural folds. (TIFF) [file pgen.1009765.s007.tiff]

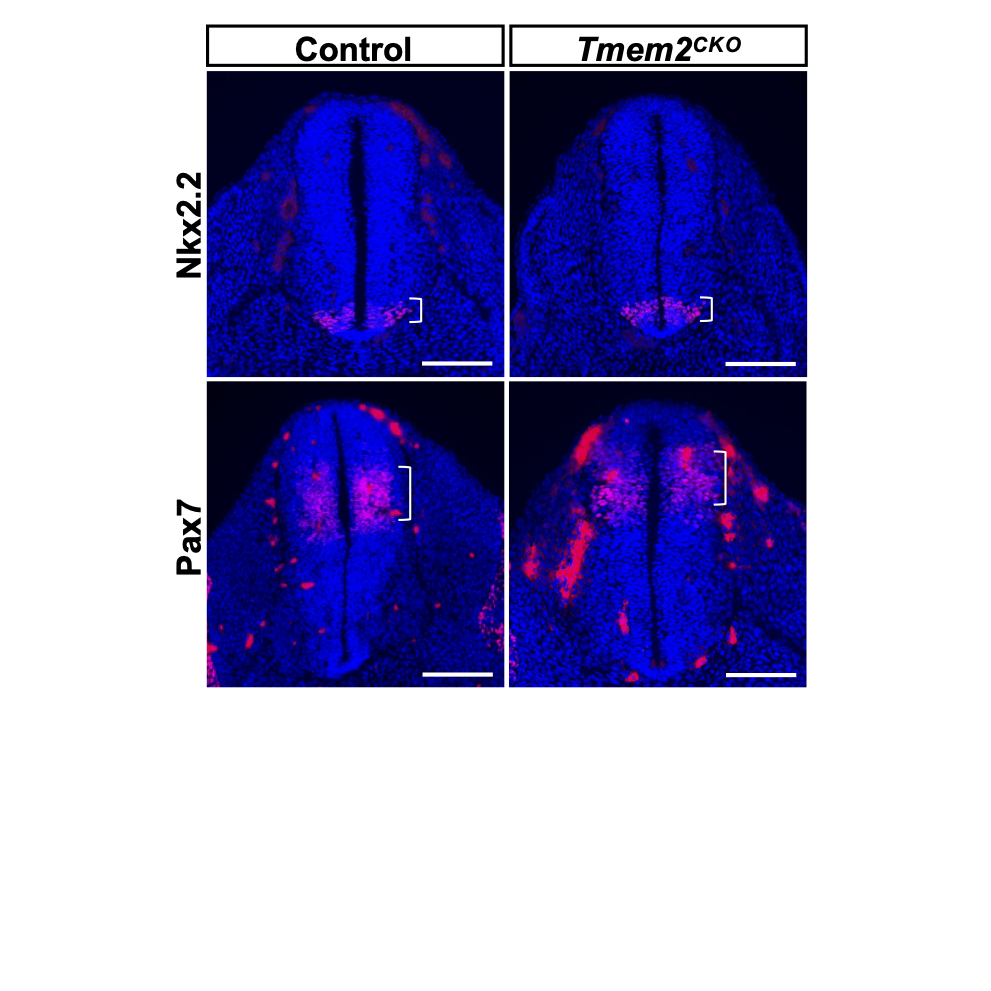

Supplement: S8 Fig — Transverse sections of the neural tube at the upper trunk level of control and Tmem2CKO embryos at E10.5 were labeled with anti-Nkx2.2 (a ventral marker) or anti-Pax7 (a dorsal marker) antibody. The overall patterning of the neural tube is not altered in Tmem2CKO mice. Scale bars, 500 μm. (TIFF) [file pgen.1009765.s008.tiff]

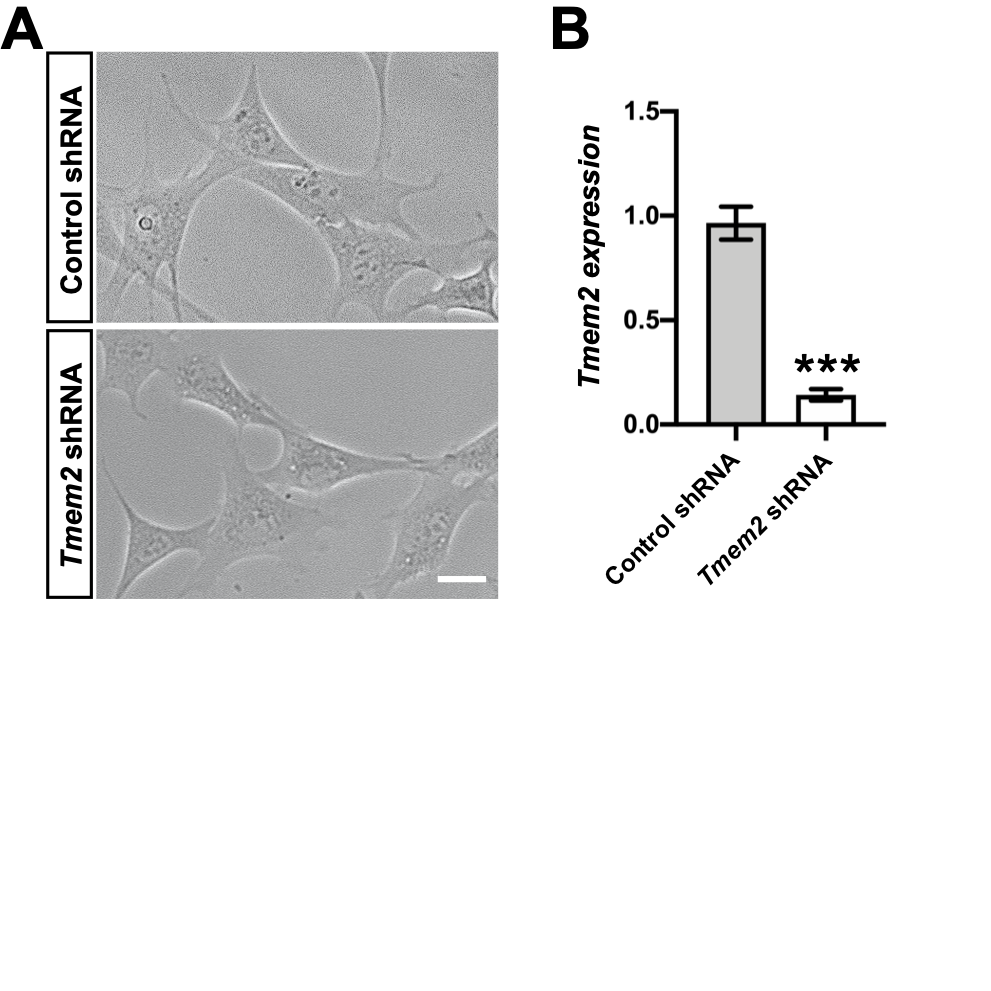

Supplement: S9 Fig — (A) Representative images of Tmem2-depleted and control O9-1 cells cultured on a regular culture dish (left). (B) Expression of Tmem2 in these cells was evaluated by qPCR, with Gapdh as an internal control for normalization (bar graph). Means ± SD (n = 5) are shown as horizontal bars. ***p < 0.001 by unpaired Student’s t-test. Scale bar, 5.0 μm. (TIFF) [file pgen.1009765.s009.tiff]

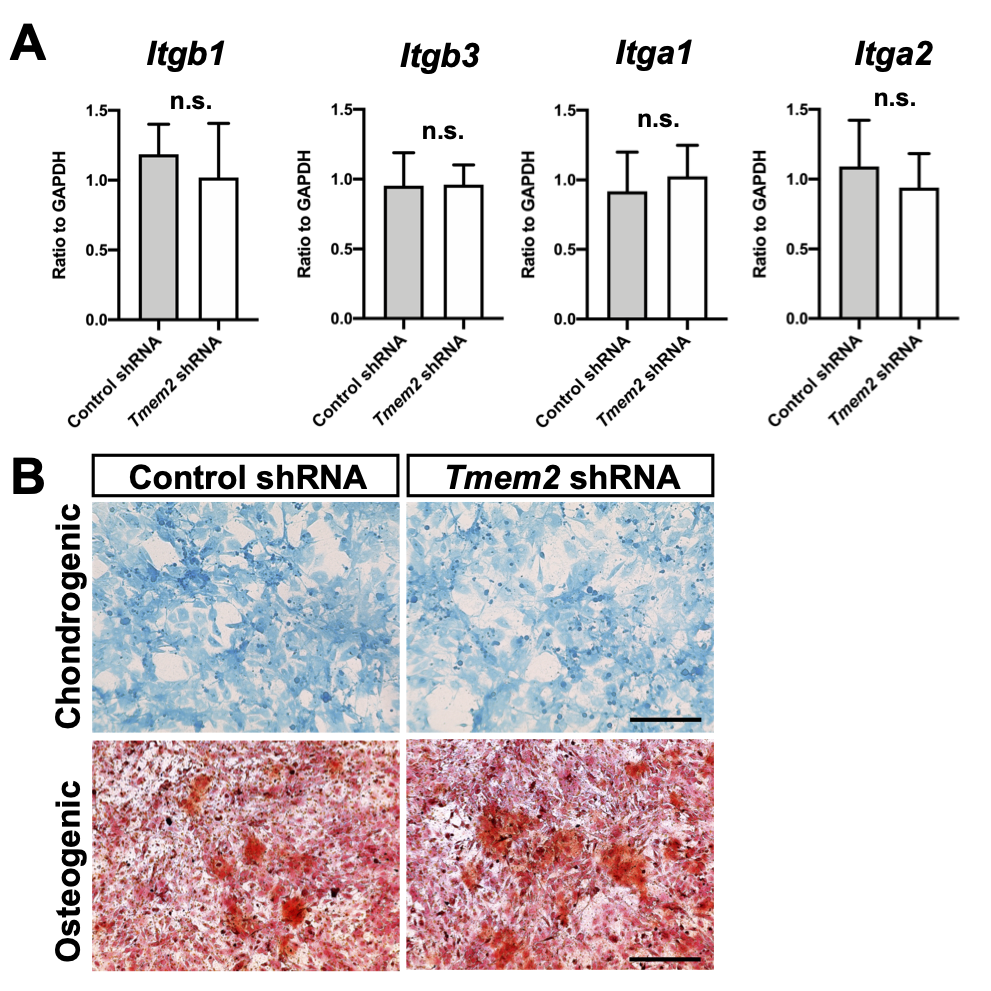

Supplement: S10 Fig — (A) qPCR analyses of integrin α1 (Itga1), α2 (Itga2), β1 (Itgb1) and β3 (Itgb3). Data represent the mean ± SD (n = 3). n.s., not significant by Student’s t-test. (B) Images show chondrogenic (alcian blue staining) and osteogenic (alizarin-red staining) differentiation of control (Control shRNA) and Tmem2-depleted (Tmem2 shRNA) O9-1 cells. Scale bars, 200 μm. (TIFF) [file pgen.1009765.s010.tiff]

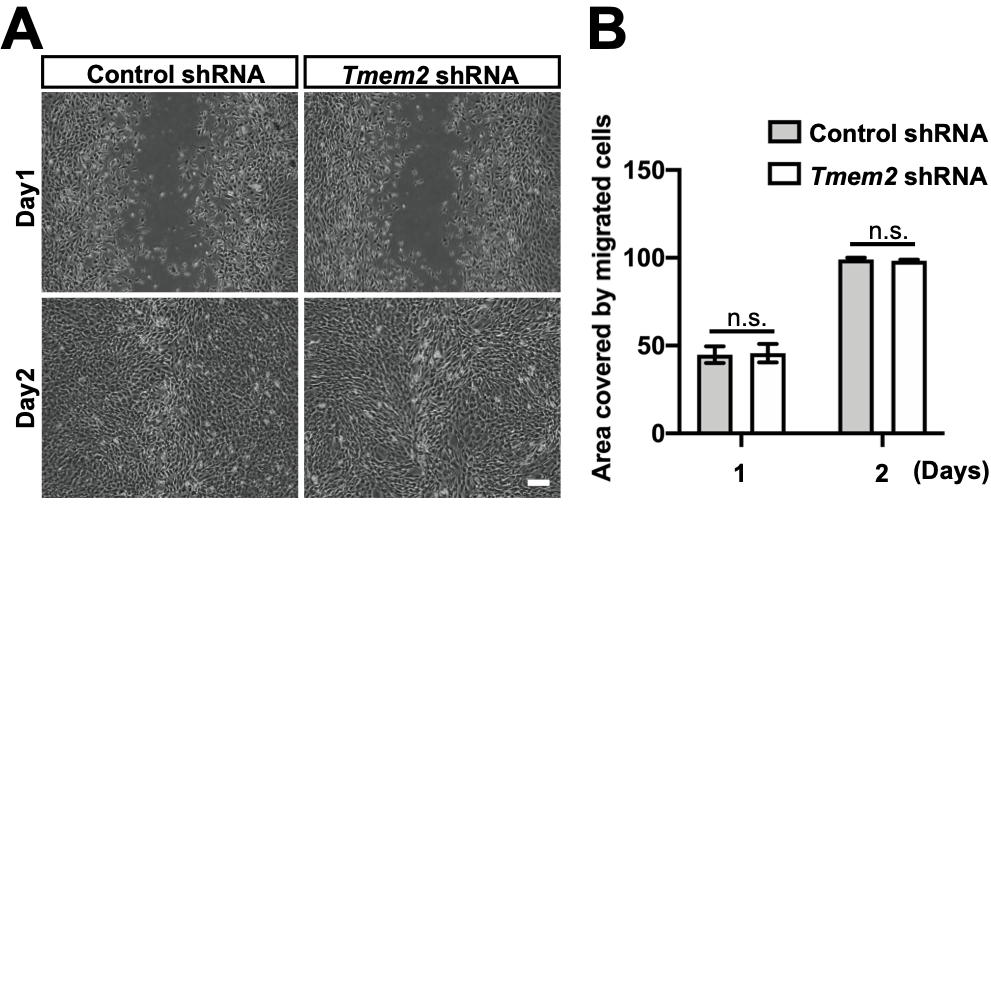

Supplement: S11 Fig — (A) Representative images of migration of control (Control shRNA) and Tmem2-depleted (Tmem2 shRNA) O9-1 cells into a cell-free gap on low molecular weight HA/Col1 mixed substrates. Panels show images after a 24 or 48 h incubation. (B) Quantitative analysis of cell migration. Data represent the mean ± SD of the gap area covered by migratory cells relative to the area of the original gap (n = 3 per condition). n.s., not significant by two-way ANOVA with Bonferroni’s multiple comparison test. Scale bar, 200 μm. (TIFF) [file pgen.1009765.s011.tiff]

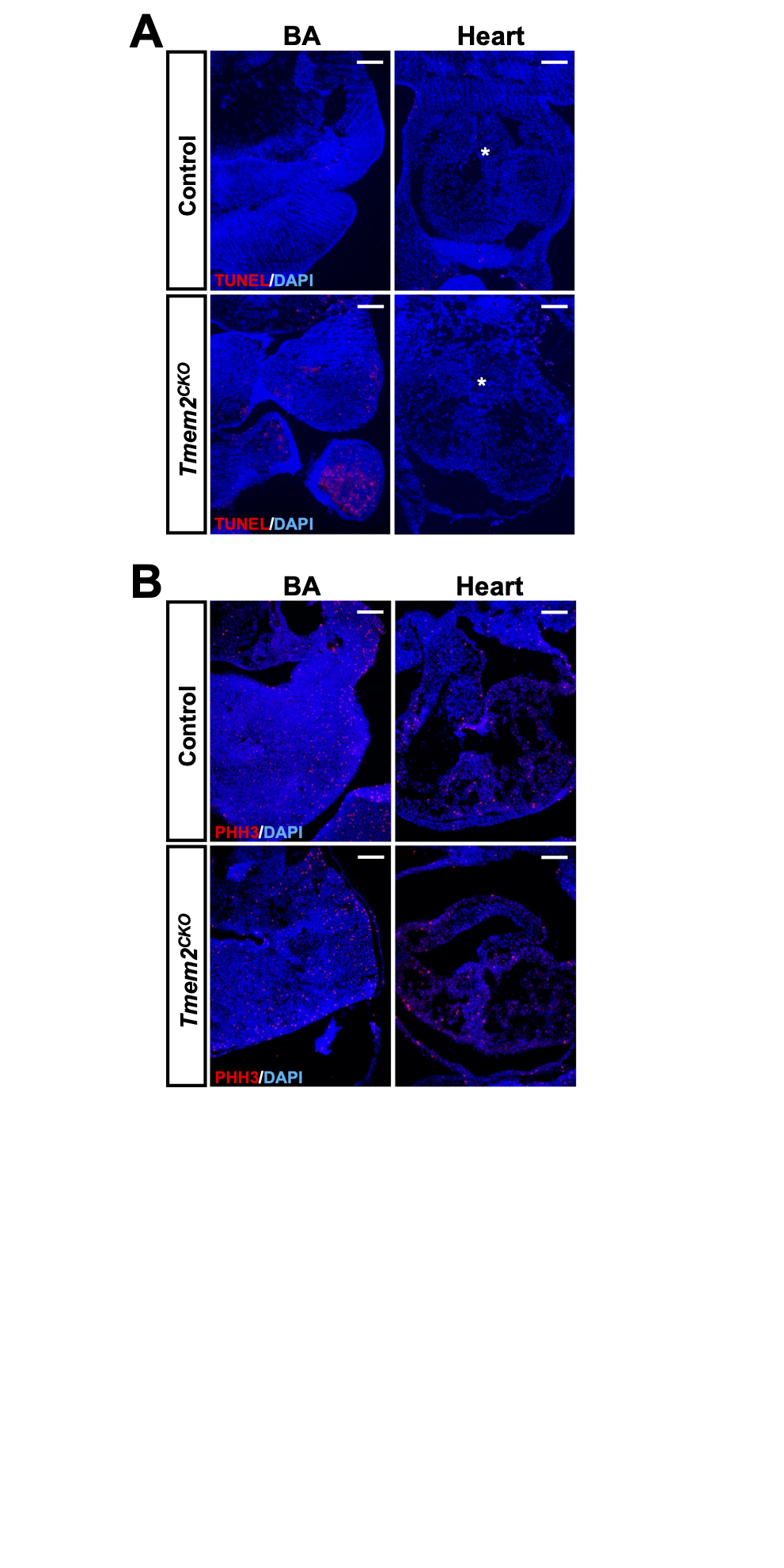

Supplement: S12 Fig — (A) Apoptotic cells were detected using TUNEL staining. Tmem2CKO embryos at E12.5 exhibit an increased number of TUNEL-positive cells in the branchial arches (BA). Asterisk indicates the outflow tract region. (B) Transverse sections of Tmem2CKO and control embryos at E12.5 were immunohistochemically stained with anti-PHH3 antibody and DAPI. Apoptosis is increased in the branchial arch of Tmem2CKO embryo compared to control embryos, whereas cell proliferation is not altered. Scale bar, 250 μm. (TIFF) [file pgen.1009765.s012.tiff]

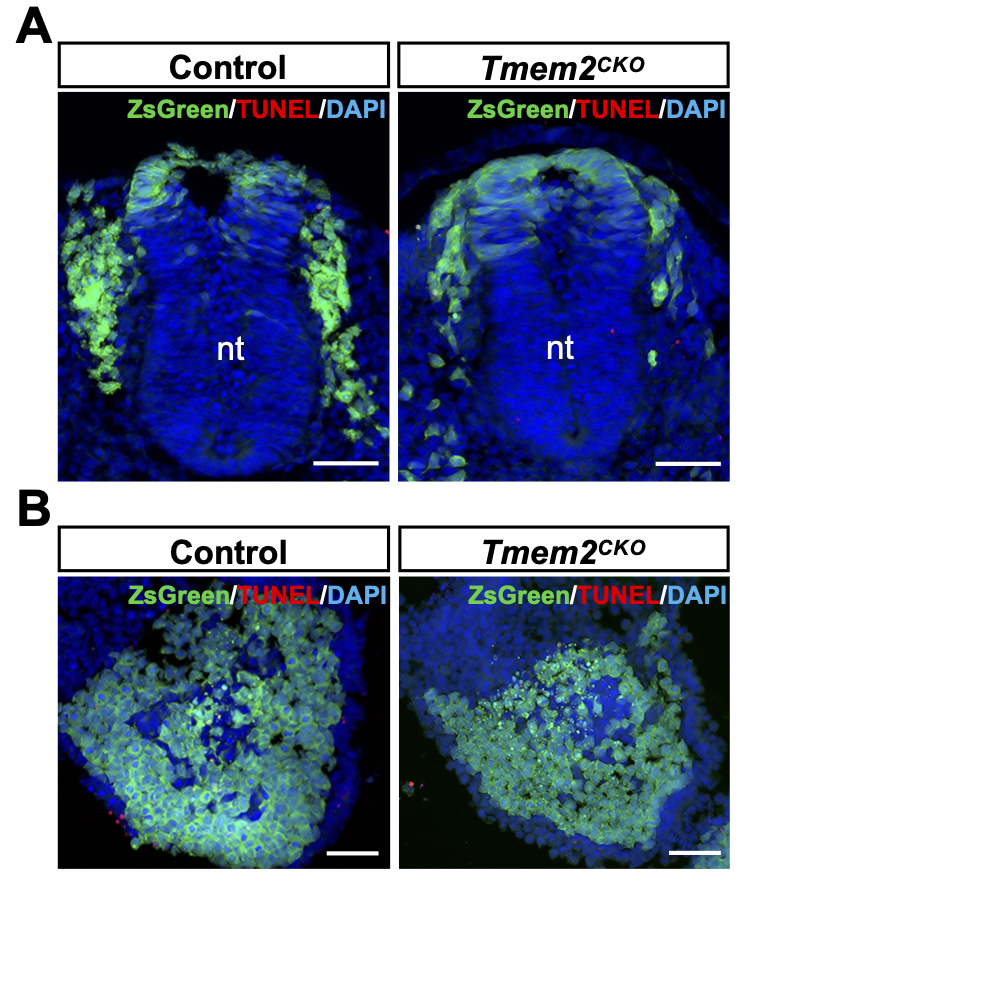

Supplement: S13 Fig — TUNEL staining (red) of the neural tube (A) and the maxillary component of the first brachial arch (B) of control and Tmem2CKO embryos at E9.0. NCCs are labeled by ZsGreen reporter (green). nt, neural tube. Scale bar, 150 μm. (TIFF) [file pgen.1009765.s013.tiff]
