# Supplementary material for: The cell surface hyaluronidase TMEM2 plays an essential role in mouse neural crest cell development and survival
Source: PLoS Genet. 2022 Jul 15;18(7):e1009765. doi: 10.1371/journal.pgen.1009765 (PMC9328550; doi:10.1371/journal.pgen.1009765)
Supplement: S1 Table — (DOCX) [file pgen.1009765.s014.docx]

S1 Table. Primer sequences for PCR genotyping of mice

| *Tmem2^flox^* forward | 5’-ATCTTGGCTTGGGTTGATGAAG-3’ |
| --- | --- |
| *Tmem2^flox^* reverse | 5’-AGGAAACCCATGCAAGCCTAC-3’ |
| *Tmem2-FLAG^KI^* forward | 5’-CAGTTTCTCCCGTTGCAGAT-3’ |
| *Tmem2-FLAG^KI^* reverse | 5’-ACAGGAAAATGGTTCCATCG-3’ |
| ZsGreen wild-type forward | 5’-AAGGGAGCTGCAGTGGAGTA-3’ |
| ZsGreen wild-type reverse | 5’-CCGAAAATCTGTGGGAAGTC-3’ |
| ZsGreen transgene forward | 5’-GGCATTAAAGCAGCGTATCC-3’ |
| ZsGreen transgene reverse | 5’-AACCAGAAGTGGCACCTGAC-3’ |
