# Supplementary material for: The cell surface hyaluronidase TMEM2 plays an essential role in mouse neural crest cell development and survival
Source: PLoS Genet. 2022 Jul 15;18(7):e1009765. doi: 10.1371/journal.pgen.1009765 (PMC9328550; doi:10.1371/journal.pgen.1009765)
Supplement: S2 Table — (DOCX) [file pgen.1009765.s015.docx]

**S2 Table. Sequences of crRNA and the inserted FLAG-encoding DNA fragment used for the creation of the *Tmem2-FLAG* knock-in allele (*Tmem2-FLAG^KI^*).**

| crRNA | 5’-GAGACTTGACCTGTTACAGC-3’ |
| --- | --- |
| Inserted DNA | 5’-AGGATTACAAGGATGACGACGATA-3’ |
