# Supplementary material for: The cell surface hyaluronidase TMEM2 plays an essential role in mouse neural crest cell development and survival
Source: PLoS Genet. 2022 Jul 15;18(7):e1009765. doi: 10.1371/journal.pgen.1009765 (PMC9328550; doi:10.1371/journal.pgen.1009765)
Supplement: S3 Table — (DOCX) [file pgen.1009765.s016.docx]

S3 Table. Primer sequences for qPCR.

| *Itgb1* forward | 5’-TGCCTACAACTCTCTTTCTTC-3’ |
| --- | --- |
| *Itgb1* Reverse | 5’-TGGTTTCAGACTCCTTATTTG-3’ |
| *Itgb3* forward | 5’-TGGAAGAGCCTGAGTGTC-3’ |
| *Itgb3* Reverse | 5’-CGGTAGGTGATATTGGTGAAG-3’ |
| *Itga1* forward | 5’-CACCTTTCAAACTGAGCCCGCCA-3’ |
| *Itga1* Reverse | 5’-GCTGCCCAGCGATGTAGAGCACAT-3’ |
| *Itga2* forward | 5’-TGGGCAAGTGCTATGTGCGTGGCA-3’ |
| *Itga2* Reverse | 5’-TCTGGGTGAAGCCGCCGCTGGT-3’ |
