# Supplementary material for: The cell surface hyaluronidase TMEM2 plays an essential role in mouse neural crest cell development and survival
Source: PLoS Genet. 2022 Jul 15;18(7):e1009765. doi: 10.1371/journal.pgen.1009765 (PMC9328550; doi:10.1371/journal.pgen.1009765)
Supplement: Attachment — Submitted filename: Responses_Tmem2 paper_0215 YY final.docx [file pgen.1009765.s017.docx]

We thank for constructive comments by the reviewers. We have performed a number of additional experiments and analyses to address the raised concerns. This revised version now includes a number of new data, which are presented as an additional panel in the main figures (Fig. 5F) and as six new Supplementary Figures (S6–10, S12). We believe that these new data address essentially all concerns raised on the original manuscript. The following is our point-by-point responses. Changes made on the text are also marked by blue fonts in the main text file.

**Response to Reviewer 1**

*1. One concern is that it is not clear which cells express TMEM2. It would be helpful to use double-labeling assays to determine which other cells express tmem2. Are other neuroectodermal derivatives also positive?*

We have previously reported TMEM2 expression pattern in E10.5 embryos by *in situ* hybridization, which demonstrated robust *Tmem2* mRNA expression in developing brain (Yamaguchi et al., *Matrix Biol*. 78/79:139-146, 2019). To further respond to this comment requesting an analysis by double-staining, we examined TMEM2 expression using the *Tmem2-FLAG^KI^* mice (which are developed and used in the current study). Double-immunostaining for nestin and TMEM2 in these mice demonstrates colocalization of immunoreactivities of nestin and TMEM2 (Supplementary Figure S6). This new result is in consistent with our previous results by *in situ* hybridization mentioned above, and affirms the expression of TMEM2 in the neuroectoderm.

*2. The cell death studies are interesting but would benefit from a more detailed examination. Is there evidence that neural crest cells themselves are undergoing apoptosis in situ or only crest derivatives? Again, double labeling would be helpful*

To address this issue, we bred Z/EG reporter embryos in *Tmem2* conditional knockout (i.e., *Wnt1-Cre;Tmem2^flox/flox^;Z/EG*) and wild-type (i.e., *Wnt1-Cre;Tmem2^wt/wt^;Z/EG*) backgrounds, in which we analyzed NCC apoptosis at E9.0 by TUNEL staining. The results are shown in Supplementary Figure S12. Unlike in the craniofacial neural crest derivatives at E12.5 (see Fig. 6), there is little increase at this stage in the number of TUNEL/ZsGreen double-positive cells in mutant embryos both in the migration pathway and in the craniofacial tissue. These results demonstrates that TMEM2 deficiency does not lead to apoptosis in migrating NCCs and NCCs just arrived at the craniofacial tissue. Instead, TMEM2 is required for the survival of post-migratory NCCs in the facial mesenchyme.

*3. One question that arises from these findings is whether hyaluronan plays a role in neural crest cell differentiation. Although migration phenotypes are clear, how would loss of tmem2 influence differentiation if the crest cell line is challenged to differentiate into different neural crest lineages?*

As suggested by this comment, we examined the effects of TMEM2 deficiency on the osteogenic and chondrogenic differentiation of TMEM2-deficient and control O9-1 cells. As shown in Supplementary Figure S9B, there are no differences on the osteogenic and chondrogenic differentiation potential between control and TMEM2 deficient O9-1 cells.

*4. Work from Roberto Perris and others suggest that progenitor cell migration may require HA synthesis. The authors should therefore discuss the possibility that HA degradation products generated by Tmem2 or other HA-catabolizing proteins, as opposed to the loss of higher molecular weight HA, may signal neural crest cells to initiate emigration from the dorsal neural tube and/or contribute to other phenotypes identified in the CKO mice.*

As suggested, a new paragraph has been added to *Discussion*, as follows (page 28):

*"It has been shown that short HA fragments and oligomers are biologically active and modulate cell signaling and behavior, including motility and migration [59, 60]. TMEM2 has been suggested to play a role in the generation of such biologically active HA fragments [61, 62]. Therefore, while our current data demonstrate the critical importance of the role for TMEM2 in NCC-matrix interactions, it cannot be ruled out that TMEM2 may also be involved in the regulation of NCC behavior via the generation motility-stimulating HA fragments. Another remaining issue is the role of HA synthesis in NCC development [63]. Inactivation of HA synthesis has been reported to result in craniofacial abnormalities [6, 7]. It is curious why both the removal and accumulation of HA cause at least superficially similar craniofacial phenotypes. Nevertheless, it is possible that the level of HA needs to be tightly regulated in a narrow range for normal development of neural crest and their derivatives. Research in integrin-mediated cell migration has demonstrated such an effect — both excessive and insufficient adhesion impedes cell migration [64, 65]. HA may exert similar effects on cell migration in the context of NCC migration."*

**Response to Reviewer 2**

*1. Tmem2 is definitely required for NCC development. And it is involved in NCC emigration, cell death. The authors introduce the mutant dies with craniofacial and cardiovascular abnormalities. Craniofacial abnormalities would be explained by decreased number of NCCs and cell death. But how do the authors explain about cardiovascular abnormalities?*

As we emphasized in the *Introduction* of the original manuscript, the primary research interest of our group is craniofacial development, and this study is intended to focus primarily on the role of this novel hyaluronidase in the process. Although NCCs play important roles in both craniofacial and cardiac development, there are significant differences in the contribution of NCCs in these developmental processes — Neural crest cells that contribute to the outflow tract of the heart ("cardiac neural crest") comprises a population with distinct developmental origin and biological properties. Accordingly, the analysis of the cardiac neural crest cells and their involvement in the outflow tract formation requires a skill set and expertise that are quite different from that for the analysis of craniofacial development. Moreover, it is rather rare that in depth analysis is performed on two different NCC subpopulations in a single study. For example, our PubMed search identified 15 PLOS Genetics papers since 2008 that have "neural crest" in the title; Essentially all these papers focused only either on cranial or cardiac neural crest development. We therefore believe that the thorough analysis of the role of TMEM2 in cardiovascular development should be conducted in a separate study. Thus, in the context of heart development, we decided to limit the current paper to the presentation of phenotypic data. However, if the reviewer (and the editor) deems it counterproductive, we will remove the data on heart development altogether.

*2. Since TMEM2 is ubiquitously expressed and consistently required, neural crest cells are constantly affected by excess hyaluronan in the mutant. After NCC migration, excess hyaluronan must affect the space for the cells, therefore, reduced number of neural crest cells in the trigeminal ganglion is reasonable. If there is not enough physical space, the number of the cells should be reduced. Do the dorasal* [sic] *ganglia show cell death? If not, why is the cell death observed in craniofacial mesenchyme?*

We found there was no significant difference in the number of apoptotic cells in DRGs between control and mutant embryos. Although the present study does not offer a mechanistic explanation for the different apoptotic response between craniofacial tissues and DRGs, a possible reason is the distinct cellular composition and the amount of ECM between DRGs and craniofacial tissues. While DRG is composed mainly of neural cells and contain little stromal components, the craniofacial tissue is composed primarily of mesenchymal cells and rich in ECM. We speculate that these differences in tissue properties have significant influence on the susceptibility of NCCs to apoptosis. Based on our intention to focus this paper on craniofacial development, we chose not to include these data and speculation in the revised manuscript.

*3. In neural crest development, after the commitment there is a step of epithelial-mesenchymal transition, then the cells delaminate. The word delamination might include the step of epithelial-mesenchymal transition, however, epithelial-mesenchymal transition is supposed to be an important step for neural crest appearance in general. Therefore, neural crest development process should be carefully explained. In addition, in the mutant does epithelial-mesenchymal transition of NCCs complete?*

According to this suggestion, we performed an analysis of the expression of Snail1 in control and mutant embryos at E8.5. This analysis demonstrates no significant difference in EMT and NCC specification between control and mutant embryos (Supplementary Figure S7A). We also show that the expression pattern of Pax3 and Pax7 is not affected in the neural tube of *Tmem2*-deficient embryos (Supplementary Figure S7B). Together, these new data demonstrate that loss of TMEM2 does not affect EMT or NCC specification within the neural tube.

*4. It is also important to describe the definition of embryonic development stage. When is the day 0?*

The presence of a vaginal plug in the dam is defined as E0.5 (according to Kaufman, M.H. The atlas of mouse development. Third edition p. 525. Academic Press; San Diego: 1999.

*5. The authors observed the domain occupied by Z/EG positive cells within the dorsal neural tube at E10.5. Neural crest cells arise at the border of neural plate and the surface ectoderm. Does Sox10 expression pattern in the mutant show the NCCs migrate within the neural tube towards ventral side?*

As shown in Fig. 4C, no aberrant migration of Sox10-positive cells within the neural tube was detected in mutants.

*6. In addition, the role of Sox9 and Sox10 expression in NCCs should be clearly defined.*

According to your suggestion, we added the following sentences in the revised test, as follows (page 15):

*"In mice, Sox9 is the first Group E Sox genes to be expressed within the neural crest, whereas Sox10 expression is initiated in NCCs as they dissociate from the neural tube and continues during NCC migration [38-40]."*

*7. What is the rationale to use the substrate for O9-1 cells to mimic the environment of emigration of NCCs?*

The general rationale for the adopting this model was described in the original manuscript ("*To model NCCs emigration into HA-rich tissues surrounding the neural tube, we performed a wound healing-type migration assay on mixed substrates consisting of type I collagen (Col1) and HA (average molecular weight 1200~1400 kDa). This in vitro model allows us to mimic the emigration of leading edge NCCs into the HA-rich interstitial ECM.*").

More specifically, we wanted to examine the behavior of migrating cells encountering the boundary of HA-rich ECM, based on the fact that type I collagen and HA are the abundant in ECM surrounding the neural tube (Perris et al. *Development* 113:969-984, 1991). The reason we chose 2D, rather than 3D, substrates is that well-established 3D models appropriate for this study are not available. Although frequently used to analyze cell migration in 3D matrices, Matrigel is not appropriate for this study, as it does contain little HA. Meanwhile, pure HA gel is anti-adhesive and inclusion of adhesive proteins, such as type I collagen, into gels is necessary. However, composite collagen-HA gels that have a uniform gelation property is difficult to prepare consistently. In comparison, the 2D substrate model has been used in our previous paper, and its usefulness for the analysis of migratory behavior of cells at the boundary of HA-rich environment has been fully validated (Irie et al. *J. Biol. Chem.* 296:100481, 2021). Together, although it may not be a perfect model for mimicking NCC emigration into HA-rich ECM surrounding the neural tube, we believe that the design of our *in vitro* migration experiment is justified.

*8. In Fig. 1, Trem2 is expressed in the dorsal part of the neural tube. In mice cranial neural crest cells appear and start migration before neural tube closure. In this image it is not clear which level of the transverse section is shown.*

We inserted the line in the image to clarify the level of the transverse section (Fig. 1A).

*9. In some of the images, the lettering is too big to understand the phenotype.*

In the relevant images, the size of the lettering has been reduced, as suggested.

*10. In Fig. 1C, legend says “Mild exencephaly-like defects”. Since the authors observed the fetus, it should be clearly mentioned. Or does “exencephaly-like” indicate another condition?*

We revised the word to "Mild exencephaly" in the legend.

**Response to Reviewer 3**

*Major Issues*

*1. The connection to cell emigration defect is not well supported especially in light of very significant apoptosis of the mutant crest cells. It is unclear when apoptosis initiates, the data are shown only for E12.5 but how about earlier? If there is earlier apoptosis it would explain why there are fewer Sox positive cells (cell death/survival versus migration defects – less cells surviving hence less cells will emigrate, time kinetics of cell death will be crucial in this regards). it will also be important to show with double-staining that the apoptotic cells are Sox9 and Sox 10 positive.*

To address these concerns, we prepared a new set of the tissue sections from control (*Wnt1-Cre;Tmem2^wt/wt^;Z/EG*) and mutant (*Wnt1-Cre;Tmem2^flox/flox^;Z/EG*) embryos at E9.0. In these sections, we examined the presence of apoptotic NCCs by the TUNEL staining. This analysis demonstrates that there are no TUNEL/ZsGreen double-positive migrating NCCs in either control or mutant embryos (Supplementary Figure S12A). In addition, there is little difference in the number of TUNEL positive cells in the facial mesenchyme between mutant and control embryos (Supplementary Figure S12B). Thus, these results indicate that TMEM2 deficiency do not significantly affect the survival of NCCs neither during migration nor in early (i.e., E9.0) facial NCC derivatives. Instead, the effect of TMEM2 on the survival of NCCs manifests in post-migratory cells at later stages (i.e., E12.5). We consider that these additional data support our model that TMEM2 deficiency induces apoptosis in NCCs at later stages in the craniofacial tissue, but not in migrating NCCs. Accordingly, the following sentences were added in *Discussion* (page 23):

*"The number of TUNEL-positive cells in migratory NCCs are not altered by Tmem2 deficiency, either (S12A Fig). Moreover, there is little difference in the number of TUNEL/ZsGreen double-positive cells between mutant and control embryos in E9.0 craniofacial tissues (S12B Fig), indicating that apoptosis of TMEM2-deficient NCCs occurs after the arrival at the craniofacial tissue."*

*1 (cont'd). Do you see cell death in vitro and can this explain the inability to create a wound in your would healing assay*

It is not very clear what this comment ("*the inability to create a wound*") implies, since the relevant experiment did not examine the *creation* of a wound. Rather, it analyzed the ability of control and *Tmem2* knockdown cells to *close* a wound. Also, the assay was intended to mimic NCC emigration, not NCC cell death observed in craniofacial tissues.

In any event, we have never observed any significant apoptosis of O9-1 cells in these assays (in both control and knockdown cells). These observations is apparently consistent with the above mentioned *in vivo* results with *Wnt1-Cre;Tmem2^wt/wt^;Z/EG* and *Wnt1-Cre;Tmem2^flox/flox^;Z/EG* embryos that there are no TUNEL/ZsGreen double-positive migrating NCCs in either control or mutant embryos.

*2. The authors don't examine the possibility that neural tube patterning may be affected in the mutants. It would be important to provide gene expression analysis of appropriate markers. Patterning defects could be an underlying reason for some of the later phenotypes especially in Figure 4B demonstrating misfolded midbrain.*

We performed additional analyses to address this issue. According to this suggestion, we examined the patterning of the neural tube in control and mutant embryos at E10.5. Specifically, embryos were immunolabelled for Pax7, a marker for the dorsal neural tube, and Nkx2.2, a marker for the ventral neural tube. As shown in Supplementary Figure S8, there were no apparent changes in the staining pattern of these markers, indicating that the patterning the neural tube is not altered in the absence of TMEM2.

*3. It is interesting that the hearts do not show apoptosis - it would be important to show whether the localization of vinculin remains affected. If no, it is feasible that vinculin defects in the crest are secondary to apoptosis – this is crucial aspect to address – what comes first, survival defect then focal adhesion lesions or vice versa. If hearts have defects in vinculin localization without apoptosis, this could serve as an important control for the former. In this regard, would be powerful to compare these phenotypes to well-characterized crest cell migration defect models.*

As stated in our response #1 to Reviewer 2, the focus of the current study is to determine the role of TMEM2 in craniofacial development, and the elucidation of the role of TMEM2 knockout on other tissues is an auxiliary issue. Please refer to the response #1 to Reviewer 2 for the detailed discussion regarding our standpoint on this matter. Altogether, we consider it scientifically more prudent to perform detailed analysis of cardiac NCCs in an independent project in the future, not as an addition to the current manuscript. We hope for the reviewer's understanding in our decision. If the reviewer considers — and the editor concurs — that the heart phenotype data should not be presented in the context of this study, we will remove the relevant data from the manuscript.

*4. If the major phenotype of mutant cells is apoptosis, it would be very interesting to investigate why loss of TMEM, and ectopic/abnormal maintenance of HA induces apoptosis and whether this is a specific feature of neural crest versus cardiac crest cells – these data would contribute novel insights to our current understanding.*

We concur that the mechanistic basis of apoptosis of TMEM2-deficient NCCs is an interesting issue surrounding TMEM2. However, we believe the issue needs to be addressed by an independent study focused on the cell and molecular biology of this novel hyaluronidase. As described in *Discussion*, there are currently at least two possible, mutually non-exclusive, mechanisms (i.e., anoikis and ER stress) to explain the phenotype. Thus, the mechanisms by which TMEM2 modulates apoptotic cell death could be quite complicated, potentially involving multiple pathways, which may or may not be cross-talking with each other. Addressing this issue will certainly require in depth analysis of multiple signaling pathways involved in these cellular responses. Again, we believe an independent, detailed study at the cell and molecular biological level is required to obtain a conclusive answer to this important question of broad significance.

*5. Similarly, if the authors claim migration as the key defect (no evidence of cell death in emigrating cells or earlier), collective polarized migration is well described for the crest cell and should be investigated in vivo with appropriate markers and imaging. Just showing less Sox+ cells is not sufficient to claim emigration defects.*

We performed additional experiments to address this issue. In collective migration, N-cadherin plays a key role in mediating adhesion of migrating. Thus, we analyzed the localization of N-cadherin on the surface of migrating NCCs. Specifically, sections of control (*Wnt1-Cre;Tmem2^wt/wt^;Z/EG*) and mutant (*Wnt1-Cre;Tmem2^flox/flox^;Z/EG*) embryos were immunostained for N-cadherin. As shown in the new Panel F of Figure 5, N-cadherin is localized on the surface of control NCCs undergoing collective migration. In contrast, cell surface association of N-cadherin is significantly reduced in mutant NCCs in migration. Thus, this result is consistent with the notion that Tmem2 deficiency directly affects the ability of NCCs to migrate from the neural tube to their target tissues.

*6. The paper would benefit from a more thorough description of existing literature about HA and NCC migration. While mentioned briefly in the introduction, the reader may not fully appreciate that HA production, as well as catabolism as shown in this paper, is absolutely critical for proper NCC migration and the development of NCC-derived structures. Related to this, the authors say that HA is a barrier against NCC migration in vitro (line 390). However, it is my understanding that HA is pro-migratory for NCCs in vivo—Has1 and Has2 are both needed for proper NCC migration in Xenopus embryos (Casini, Nardi, and Ori, 2012), while versican acts as a barrier against NCC migration (Landolt et al., 1995). While the data in figure 5 supports your argument, I think it is important to give the full picture of the in vivo data that we have about NCC migration and the ECM. Similarly, when Has2 is conditionally knocked out in cranial NCCs, mutant mice have cleft palate due to diminished palatal shelves that don’t move and fuse properly (Yonemitsu, Lin, and Yu 2020).*

According to this suggestion, we revised the second paragraph of *Introduction* as follows (page 5):

*"Hyaluronan (HA) is a non-sulfated glycosaminoglycan widely distributed in the ECM of a variety of tissues. The importance of HA in the development of NCC-derived tissues is illustrated by the observation that disruption of HA synthesis by means of genetic ablation of the Has genes, which encode hyaluronan synthases, leads to defects in NCC-derived tissues. In mice, Has2 ablation results in mid-gestation (E9.5-10) lethality due to severe defects in endocardial cushion formation [3, 4]. Defects in these cardiac structures are often associated with the abnormal development of a subpopulation of cranial NCCs [5]. Cranial NCC-targeted conditional Has2 knockout mice also exhibit these defects in formation of the NCC-derived craniofacial structures [6]. In Xenopus, mutations in hyaluronan synthase genes (has1 and has2) result in impairments in NCCs migration and craniofacial defects [7]. It has also shown that the HA-binding proteoglycans aggrecan and versican exerts inhibitory effects on NCCs migration [8, 9]."*

Also, a new paragraph has been added to *Discussion* (same as our response #4 to Reviewer 1):

*"It has been shown that short HA fragments and oligomers are biologically active and modulate cell signaling and behavior, including motility and migration [59, 60]. TMEM2 has been suggested to play a role in the generation of such biologically active HA fragments [61, 62]. Therefore, while our current data demonstrate the critical importance of the role for TMEM2 in NCC-matrix interactions, it cannot be ruled out that TMEM2 may also be involved in the regulation of NCC behavior via the generation motility-stimulating HA fragments. Another remaining issue is the role of HA synthesis in NCC development [63]. Inactivation of HA synthesis has been reported to result in craniofacial abnormalities [6, 7]. It is curious why both the removal and accumulation of HA cause at least superficially similar craniofacial phenotypes. Nevertheless, it is possible that the level of HA needs to be tightly regulated in a narrow range for normal development of neural crest and their derivatives. Research in integrin-mediated cell migration has demonstrated such an effect — both excessive and insufficient adhesion impedes cell migration [64, 65]. HA may exert similar effects on cell migration in the context of NCC migration."*

*6 (cont'd). What explanation do you propose for both a hyaluronidase (TMEM2 and some Hyal2 null—Muggenthaler et al., 2017) and an HA synthase having the same phenotype when knocked out?.*

We notice that the experimental approaches of these studies are different (e.g., constitutive KO vs conditional KO) and the severity of craniofacial defects are quite distinct. For example, the craniofacial phenotype of *Hyal2^–/–^* mice reported by Muggenthaler et al. is obviously much milder than that of our *Tmem2* CKO mice — *Hyal2^–/–^* mice are born alive, and moreover, the fusion of frontonasal processes and the formation of other craniofacial bones occurs largely normal. Lack of fusion is reported only for the vomeronasal organ and primary palate. Importantly, in *Hyal2^–/–^* mice, the *Hyal2* gene is ablated not only in cranial NCCs but also other cell types that form the craniofacial structure. Given these differences, it is possible that the phenotype in *Hyal2^–/–^* mice may not be solely neural crest-dependent. Considering all these complications, we feel it is difficult to provide an explanation based only on the information available in literatures.

On the other hand, both *Tmem2* CKO and *Has2* CKO mice (Lan et al., *J. Dent. Res.* 98:1367-1375) were generated using the same Wnt1-Cre driver, and why these two knockouts develop similar craniofacial defects cannot easily be explained at present. Nevertheless, one intriguing possibility is that the level of HA needs to be tightly regulated in a narrow range for normal development of neural crest and their derivatives. Research in integrin-mediated cell migration has demonstrated that efficient cell migration requires appropriate levels of adhesion strength — both excessive and insufficient adhesion impedes cell migration (see e.g., DiMilla et al., *J. Cell Biol*. 122:729-737, 1993; Gardel et al., *Ann. Rev. Cell Dev. Biol*. 26:316-333, 2010). HA may exert similar effects on cell migration *in vivo* — i.e., both excessive accumulation and loss of tissue HA inhibit normal NCC migration. This possibility is discussed in the new paragraph in *Discussion* mentioned above (see #6).

*6 (cont'd). Does the importance of both HA production and HA degradation suggest that it is in fact HA fragments that are key for NCC migration? This could be tested in your in vitro system, where you could make separate Col1/HA substrates with high molecular weight HA or with HA fragments, and see if the migration defect exhibited by Tmem2 shRNA cells is rescued when grown on substrate with HA fragments.*

We performed an additional study suggested by this comment. Specifically, the wound healing assay with O9-1 cells was performed with Col1/HA composite substrate with low molecular weight (LMW) HA. Results demonstrate that there are no differences in migration between control and *Tmem2* knockdown O9-1 cells on these substrates (Supplementary Figure S10). Thus, this result indicates that the inhibitory effect of HA on NCC migration requires high molecular weight HA species, and that LMW HA fragments do not exert a significant effect.

*Minor Issues*

*1. Line 29: “regulated in during development”—change to “regulated during development”*

*2. Line 60: change NSC to NCC*

*3. Lines 87-90—The authors try to connect the Has2 KO phenotypes in cardiac cushions with cranial NCCs, but with very weak evidence.*

*6. Line 395: “stems” instead of “steams”*

*10. Line 336 spelling: lineage tracing not trancing*

*11. Line 339: briefly describe what this strain is: ZsGreen (Z/EG)*

These comments have been addressed by revising the text as suggested.

*4. Figure 1b: would be better to have an image of a mutant embryo that doesn’t have tissue damage (forelimb looks like it was almost cut off, at first glance this appears to be a phenotype)*

We searched our data files for another image of a mutant embryos of the same litter. Unfortunately, no such images are available. Thus, we added a note in the legend of the figure to explain this is not a phenotype, as follows:

"*Asterisk indicates a partial tear of the right limb bud that inadvertently caused during dissection.*"

*5. Figure 1f: could quantify the cell-free spaces in the H&E images to further illustrate the difference in ECM between wild-type and mutant. Similar could possibly be done for Figure S3.*

We believe that the significant increase in extracellular spaces in mutant mice is obvious without quantification. Also, since H&E staining does not define cell contours (hematoxylin stains nuclei), it is not optimal for the quantitative analysis of the size of extracellular space. Accordingly, we chose not to perform the quantification.

*7. Have you checked if integrins are present at the same number on TMEM2 knockdown cells as on wild-type cells? If integrins were downregulated that would be an alternate explanation for lack of focal adhesions (unlikely, but good to check).*

According to this suggestion, we performed RT-PCR analysis on integrins. The predominant integrin isoform expressed in neural crest cells (including O9-1 cells) is β1, while α5, α6, and β3 have also been shown to be expressed (Ishii et al., *Stem cells Dev.* 21:3069-3080, 2012; Barriga et al., *Nature* 554:523-527, 2018; Strachan and Condic, *J. Cell Biol.* 167:545-554, 2004; Alfandari et al., *Dev. Biol.* 260:449-464, 2003). As shown in Supplementary Figure S9A, there are no significant differences in the levels of expression of these integrin isoforms between control and *Tmem2* knockdown cells.

*8. In vitro migration assay, can you rescue with transfection of TMEM2?*

Based on the following considerations, we did not perform rescue experiments. Since the sequence of the shRNA used for TMEM2 knockdown is highly conserved between human and mouse TMEM2, mutagenesis of synonymous mutations at multiple nucleotide residues within the targeted sequence was considered necessary to ensure its rescuing activity. We deemed that the construction of a lentivirus vector carrying such a TMEM2 cDNA, followed by the packaging of virus particles and the production of high-titer virus preparation, would take a long time beyond the time frame allowed.

Meanwhile, we believe that our data provide ample support for the specificity of the observed shRNA effects, and therefore, that rescue experiments are not necessary. First, the shRNA used in our study was selected by The RNAi Consortium/Broad Institute based on a specialized algorithm that aims to effectively knockdown only the target gene without off-target effects and its effect has subsequently been validated in human cells. Moreover, we showed that the expression of multiple integrin genes is not affected by this shRNA (see #7 above), confirming the absence of no off-target effects on the genes that could confound the interpretation of the results. Finally, O9-1 cells transduced with this shRNA adhere to and migrate on composite Col1/low molecular weight HA substrates normally. This observation indicates that the adhesion/migration machinery of O9-1 cells is not altered by the shRNA. Moreover, because TMEM2 cannot cleave short HA species (<5 kDa; Yamamoto et al., *J. Biol. Chem*. 292:7304-7313), it also suggests that the impaired migration of knockdown cells on Col1/HMW HA substrates is dependent on the HA-degrading activity of TMEM2, not on any off-target effects.

*9. Line 309: Sox10 staining (Fig 3B) demonstrates that the number of NCCs migrating along the ventral migration pathway is reduced”. What about a pan marker of crest, as possible that total number is reduced so migrating number will also be reduced.*

Due to time constraint, we did not perform this analysis. However, we believe that our data as a whole, including those with Wnt1-Cre–dependent ZsGreen-labeling of NCCs, have firmly established the role of TMEM2 in NCC migration, even without this requested analysis.

*12. Line 404: what is the evidence that HA is being degraded here*

Dark holes/streaks in the green HA substrates represent the sites where HA is degraded by cells. Please refer to Yamamoto et al., *J. Biol. Chem*. 292:7304-7313) for the basis of the assay.

*13. Is HA accumulation also seen in the mutant hearts or neural tube specific?*

Increased tissue HA levels have also been observed in the NCC derivatives in the heart, although it is not as prominent as in the facial mesenchyme.

*14. Time kinetics of TMEM expression prior to E9, when does expression initiate? Does it coincide with NCC specification or migration?*

Our preliminary data have revealed that *Tmem2* is expressed in gastrulating embryos at as early as E7.5. (A manuscript describing this and other observations is in preparation.) We do not know the exact timing of the initiation of *Tmem2* expression. However, since *Tmem2^–/–^* embryos undergoes implantation normally, the initiation of Tmem2 expression likely occurs between implantation and gastrulation.
