# Supplementary material for: The cell surface hyaluronidase TMEM2 plays an essential role in mouse neural crest cell development and survival
Source: PLoS Genet. 2022 Jul 15;18(7):e1009765. doi: 10.1371/journal.pgen.1009765 (PMC9328550; doi:10.1371/journal.pgen.1009765)
Supplement: Attachment — Submitted filename: Responses_220518 FINAL2.docx [file pgen.1009765.s018.docx]

We are pleased to be informed that both the reviewers and the editors agree that the revisions made on the previous version of the manuscript have significantly improved our paper. Now, Reviewer 1 has no concerns, while Reviewers 2 and 3 raises several remaining issues. These remaining issues center mainly on: (a) the way in which the data were presented ("*The way how the authors present the results is too rough*."), and (b) the lack of statistical analyses on some of the data ("*… one cannot conclude significant changes without statistic*s."). To address these issues, we have revised the manuscript in three key aspects. First, the text and the presentation of data have been revised to improve the readability of the paper. For this, we consulted a developmental biologist familiar with neural crest development, and also hired a professional scientific editor to review and edit the text. Second, we reformatted figures to improve the presentation of the data. Third and most importantly, we performed quantitative analyses as suggested by Reviewer 3. These new data with statistics are now presented in Fig. 1F, Fig. 3C, and Fig. 5A. We believe that these revisions and new data should address all remaining concerns on our manuscript. The following is our point-by-point responses. Please note that, because of a number of grammatical and sentence structure edits made on the manuscript, only newly added statements/sections are marked in the text (in *blue* fonts).

**Reviewer #1:** Reviewer #1 raises no concerns.

**Reviewer #2:**

*1. The way how the authors present the results is too rough. For instance, the authors write “Fig. 3C graph shows the quantification of migrating NCCs in Tmem2CKO;Z/EG and control embryos at E10.5.”, however, neural crest cell migration completes by E10.5. Further, some of the results needs higher magnification images to understand what the authors write in the text. The authors are advised to consider which data sets clearly indicate what they want to inform to the readers.*

We appreciate these comments. Obviously, we were not careful enough in preparing the previous version due to time constraints stemming from the fact that a large number of additional experiments had been performed for the previous revision.

To address these issues and to improve the clarity of the manuscript, this revised version has been read by a developmental biologist familiar with the subject matter and further edited by a professional scientific editor. These revisions include the sentence referred above by this reviewer, so that the inaccurate use of the term (i.e., "migrating NCCs … at E10.5") is avoided (Lines 345-349). Also, as suggested by this reviewer, all figures have been revised to improve the understandability of the images. Specifically, in Fig. 2C and Fig. 4C, the size of some key images have been increased. In Fig. 1B, additional panels of high-magnification images have been added. To accommodate these new images, the original Fig. 1A (*in situ* hybridization for *Tmem2* in wild-type embryos at E8.5 and E9.0) has been moved to Supplements and combined with S1 Fig as S1A Fig. Description of figures in the main text, as well as individual figure legends, have also been revised to make the relationship between the images and their description clearer. We believe these revisions have greatly enhanced the readability of this paper.

*2. Depletion Tmem2 in OP-9 induced cell death, but not all the OP-9 cells. Large portion of craniofacial mesenchyme is neural crest origin, however, the region of cell death is limited. Is it possible to explain?*

The available data do not provide a definite explanation for this issue. However, our results (Fig. 6C, Fig. 6D, and S11 Fig) show that the sensitivity of O9-1 cells to apoptosis is dependent on the presence and the size of HA. Thus, the regional difference in the level of cell death in the craniofacial tissue may be due to the difference in the level and/or the size of HA in these regions.

*3. Line 204, the word “hereafter” is duplicated.*

Corrected.

*4. Line361-362, what does the phrase “whereas the domain occupied by Z/EG-positive cells within the dorsal neural tube is expanded in Tmem2CKO;Z/EG embryos” mean?*

The phrase was meant to state that the number of ZsGreen-positive cells within the neural tube was increased, and that the spatial distribution of ZsGreen-positive cells was expanded. To clarify the point, the relevant sentence has been revised as follows:

"*In Tmem2CKO;ZsGreen embryos, the contribution of ZsGreen-positive cells to trunk neural tube-derived dorsal root ganglia is reduced (Fig 4C, arrowheads), whereas ZsGreen-positive cells within the neural tube are increased in number and their distribution is spatially expanded ventrally (Fig 4C, bracket).*" [Lines 327-330]

**Reviewer #3:**

*This reviewer appreciates the detailed responses; however, the following points (#5 and #12) need quantifications, one cannot conclude significant changes without statistics. … Along the same lines, point #9 is important if the authors want to conclude that the number of Sox10 cells is reduced, if this is significantly reduced is has to be normalized to the total number of cells.*

Following this general comment, we have added quantitative data (with statistics) to address all these issues (i.e., Points #5, #9, and #12). See below for our specific responses regarding these new data.

***(Point #5)*** *Figure 1f: could quantify the cell-free spaces in the H&E images to further illustrate the difference in ECM between wild-type and mutant. Similar could possibly be done for Figure S3.*

Following this comment, we performed a quantitative analysis of extracellular space using a method employed in earlier studies (e.g., Senf et al., PLOS One 8:e62687, 2013). Our analysis confirmed a statistically significant difference in the size of extracellular space between *Tmem2^CKO^* and control mice. This new result is presented as a bar graph added to Fig 1F.

Since the cardiac phenotype is not the main theme of this study (this point has been discussed in our response to the previous critiques), we chose not to perform a similar analysis on the heart tissue. Accordingly, we modified the sentence describing the phenotype of the heart tissue by deleting the phrase "*with sparse mesenchyme*." The revised sentence reads as follows:

"*Consistent with the functional role for TMEM2 in cardiac NCCs, Tmem2^CKO^ embryos exhibit expanded endocardial cushions and lack of the aorticopulmonary septum in the outflow tract region (S3 Fig)*." [Lines 178-180]

***(Point #9)*** *Line 309: Sox10 staining (Fig 3B) demonstrates that the number of NCCs migrating along the ventral migration pathway is reduced”. What about a pan marker of crest, as possible that total number is reduced so migrating number will also be reduced.*

Our extensive search (PubMed, GoogleScholar) for pan neural crest cell markers that could be reliably used in mice did not identify any marker. We found that a few papers back in the 1990's used HNK-1 and P75NTR as pan-NCC markers in studies with chick embryos; however, we found these molecules have never been established as pan-NCC markers in mice. We also considered antibodies against crestin, a molecule that has been used in several papers on neural crest research in zebrafish. However, its use has been limited to research in zebrafish, and moreover, we could not find any anti-crestin antibodies with a reactivity to the mouse counterpart. Therefore, we instead used the sum of the numbers of Sox9-positive cells and Sox10-positive cells as an approximate representation of the total number of NCCs in each embryo. Specifically, the number of Sox9-positive cells and Sox10-positive cells were individually counted, and from these data, we calculated the percentage of emigrated NCCs relative to the total NCCs, which was then statistically compared between *Tmem2^CKO^* and control mice. As you see in the third bar graph in Fig. 3C, the ratio of emigrated NCCs, normalized to the total number of NCCs, is significantly reduced in *Tmem2^CKO^* embryos than in control embryos. Thus, although we could not use a pan-NCC marker as suggested by the reviewer, we believe that this new data substantiates our conclusion that the reduced number of NCCs migrating outside the neural tube in *Tmem2^CKO^* mice is primarily due to impaired emigration *Tmem2*-deficient NCCs.

Meanwhile, we realized that this and another comment ("*Along the same lines, point #9 is important if the authors want to conclude that the number of Sox10 cells is reduced, …*") by this reviewer suggest that we should be more deliberate in stating our conclusions/interpretations and also should provide more balanced discussion. Therefore, a new discussion regarding this issue has been added as follows:

"*On the other hand, our present data do not provide a definitive answer regarding a possible requirement for TMEM2 in the subsequent migration of NCCs that have emigrated from the neural tube. This process is governed primarily by a collective migration mechanism [56, 57], which does not typically rely on the formation of FAs by individual cells. Still, it is possible that the effect of TMEM2 on cell adhesion may not be limited to integrin-dependent cell-ECM adhesions. This possibility is suggested by the observation of reduced cell surface association of N-cadherin in Wnt1-Tmem2CKO;ZsGreen embryos (Fig 5E). The possible role for TMEM2 in N-cadherin–mediated cell-cell adhesion is thus an issue that needs to be addressed in order to fully understand the function of this novel hyaluronidase in NCC migration.*" [Lines 533–541]

***(Point #12)*** *Line 404: what is the evidence that HA is being degraded here*

Responding this question, we performed a quantitative analysis of the hyaluronidase activity in *Tmem2*-depleted and control O9-1 cells using the *in situ* hyaluronidase assay on substrate-coated fluorescein-labeled HA, which was cited in the manuscript (Yamamoto et al., 2017; Ref. 30). Based on the same principle as the widely used *in situ* zymography for the collagenase activity of matrix metalloproteinases, in this hyaluronidase assay, degradation of HA by cells is revealed as dark spots in a fluorescent background, and the level of hyaluronidase activity can be quantified by measuring the reduction of the fluorescence intensity under cells. We have previously reported that the level of dark spot formation correlates with the level of TMEM2 hyaluronidase activity in *in vitro* assay (Ref. 30). As shown in Fig. 5A (which is a new addition to this revised version), control O9-1 cells exhibit robust HA degrading activity. In this experiment, we also compared the hyaluronidase activity of *Tmem2*-depleted and control O9-1 cells, which shows that hyaluronidase activity is significantly reduced in *Tmem2*-depleted O9-1 cells, consistent with our assumption that TMEM2 is the main cell surface hyaluronidase in O9-1 cells.

*Finally, in the new figure (S8) (point #2) - it is important to specify whether this is cranial/caudal neural tube, since in the other figures (Fig. 2 and 3), the caudal neural tube always looks fine and it’s only the cranial neural tube where phenotypes are seen.*

The result shown in the relevant figure is on the neural tube at an upper trunk level. The legend of the figure (S8 Fig) has been revised to include this information.
